# Response terminated displays unload selective attention

**DOI:** 10.3389/fpsyg.2013.00967

**Published:** 2013-12-24

**Authors:** Zachary J. J. Roper, Shaun P. Vecera

**Affiliations:** Department of Psychology, University of Iowa, Iowa CityIA, USA

**Keywords:** perceptual load, selective attention, visual short-term memory, visual awareness

## Abstract

Perceptual load theory successfully replaced the early vs. late selection debate by appealing to adaptive control over the efficiency of selective attention. Early selection is observed unless perceptual load (*p*-Load) is sufficiently low to grant attentional “spill-over” to task-irrelevant stimuli. Many studies exploring load theory have used limited display durations that perhaps impose artificial limits on encoding processes. We extended the exposure duration in a classic *p*-Load task to alleviate temporal encoding demands that may otherwise tax mnemonic consolidation processes. If the load effect arises from perceptual demands alone, then freeing-up available mnemonic resources by extending the exposure duration should have little effect. The results of Experiment 1 falsify this prediction. We observed a reliable flanker effect under high *p-*Load, response-terminated displays. Next, we orthogonally manipulated exposure duration and task-relevance. Counter-intuitively, we found that the likelihood of observing the flanker effect under high *p-*Load resides with the duration of the task-relevant array, not the flanker itself. We propose that stimulus and encoding demands interact to produce the load effect. Our account clarifies how task parameters differentially impinge upon cognitive processes to produce attentional “spill-over” by appealing to visual short-term memory as an additional processing bottleneck when stimuli are briefly presented.

The world around us is saturated with rich visual information; however, only a small subset of this information reaches conscious awareness at a given point in time. To accomplish this feat, the visual system operates with extreme prejudice by filtering all adequate visual stimuli to a small subset of privileged information. The outcome of this judicious behavior is a cognitive process known as selective attention. So that we may optimally behave on a variable environment, selective attention finely resolves visual input in accordance with biological imperatives. Attention selects stimuli that will aid an organism’s pursuit of specific biological objectives, e.g., to locate food, to find sanctuary, to assess social relevance, to find a mate, et cetera (Most et al., 2007; Hodsoll et al., 2011). Optimal selection strategies give rise to adaptive behavior. However, this process is imperfect; sometimes attention selects more information than what is needed and sometimes less. Given a noisy input channel, on sheer probability alone it is rare that attention selects no more than desired and no less than necessary. Thus attentional control vacillates, throttling open to capture more of the environment when additional information is needed to complete a task and throttling down when narrower focus is needed to prevent distraction (Lien et al., 2010). In this fashion attention flexibly accommodates to the dynamic environment and is said to operate with a late locus when distractors command behavior, and an early locus at all other times (Yantis and Johnston, 1990; Miller ’s 1991). That selective attention operates with an arbitrary amount of tolerance at all was not immediately understood. In fact, it took more than four decades to resolve the flexible locus hypothesis.

Early selection views prevailed in the 50s and 60s with pioneering attention studies (e.g., Cherry, 1953; Neisser, 1969; Treisman, 1969). However, the consensus shifted toward late selection views in the 70s and 80s (e.g., Deutsch and Deutsch, 1963; LaBerge, 1975; Allport, 1977; Duncan, 1980). Load theory was developed in the 90s to reconcile these disparate findings by appealing to the possibility of adaptive attentional control (Lavie and Tsal, 1994; Lavie, 1995). Load theory proposes that selective attention acts early, but is more efficient under high perceptual load (*p*-Load) than low *p*-Load (see Benoni and Tsal, 2013 for a concise review of load theory).

Load theory garners support from the flanker paradigm (e.g., Eriksen and Hoffman, 1973; Eriksen and Eriksen, 1974). In the simplest version of the flanker task, a target appears in the center of a homogeneous letter array (see **Figure 1**). The target takes on one of two possible identities, each of which is mapped onto a unique response key. Whereas congruent distractors share the target’s response, incongruent distractors are mapped onto an alternate response key. The resulting trials are response congruent and incongruent, respectively. Incongruent distractors engender conflict during response selection and yield greater response time (RT) when observers must report the target’s identity (Miller, 1991). This robust RT difference reflects the flanker effect and behaviorally indicates that the flanking distractors were processed to at least the point of meaning. Significant flanker interference effects epitomize late selection processes (Yantis and Johnston, 1990).

**FIGURE 1:**
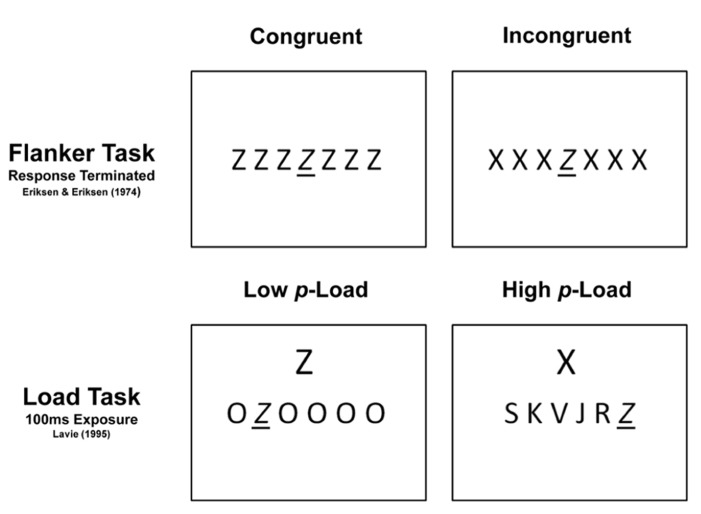
**Canonical Flanker and load tasks.** The Eriksen Flanker task, top two panels, has both congruent and incongruent conditions. In the Eriksen Flanker task, the target is always located in the center. In the load task, lower two panels, the Flanker is either above or below the stimulus array and the relevant load is determined by the relationship between the distractors in the linear array and the target; additionally, the target can appear at any one of the six linear locations. The targets in these examples (all the letter “Z”) have been italicized and underlined for distinction. Whereas the Flanker task employs response terminated displays, the load task uses brief exposure.

To address the possibility of a flexible locus of attention, Lavie (1995) revamped the flanker task by varying the difficulty to find the target. Heterogeneous distractors that resemble the target characterize high *p*-Load displays (Roper et al., 2013). Phenomenologically, these displays appear more cluttered than Eriksen and Eriksen’s (1974) flanker displays (see **Figure 1**). Lavie proposed that an early filter is only established when attentional “resources” are fully taxed, that is, when *p*-Load is high. Under all other circumstances, load theory predicted surplus “resources” mandatorily “spill-over” to process task-irrelevant stimuli. Indeed, Lavie’s results supported her predictions; significant flanker effects obtain only under low *p*-Load.

Despite the successes of *p*-Load theory, the nature of the attentional “resources” it invokes is unclear. Recent attempts to operationally define *p*-Load have successfully integrated load theory into the broader attention literature. Torralbo and Beck (2008) proposed that the load effect arises from the need to resolve low-level stimulus interactions between the target and distractors. Extending from Torralbo and Beck (2008); Lavie and Cox (1997), and Duncan and Humphreys, 1989), we demonstrated a clear role for target-distractor similarity and distractor homogeneity in the context of *p*-Load (Roper et al., 2013). Using identical stimuli in a visual search task and a canonical *p*-Load task, we discovered a close correspondence between search efficiency and flanker effect magnitude. We found that low *p*-Load displays were searched efficiently, whereas high *p*-Load displays were not. These findings suggest that load might be defined by appealing to bottom-up stimulus interactions that are known to affect visual search. However, close consideration of the typical *p*-Load task suggests that bottom-up stimulus factors do not completely account for the “resources” of *p*-Load theory.

At least two component processes are at play when an observer performs the canonical *p*-Load task: (1) feature-based attention needed to resolve inter-stimulus competition (Lavie and Cox, 1997; Torralbo and Beck, 2008; Roper et al., 2013), and (2) encoding-based processes needed to endogenously represent fleeting stimuli (Jolicœsur and Dell’ Acqua, 1999). The thrust of our current work was to closely examine specifically how exposure duration operates to bring about encoding challenges. This was done so that we may better characterize the nature of attentional “resources” – feature-based and encoding-based – that are responsible for “spill-over.”

To date, nearly all load studies have employed brief target display durations. For example, Lavie’s (1995) original work incorporated 100 ms stimulus exposure durations. Similarly, a concise but representative literature review revealed that subsequent load studies have followed suit with exposure durations ranging from 17 to 200 ms, having a modal duration of 100 ms (Lavie and Cox, 1997; Bavelier et al., 2000; Handy and Mangun, 2000; Lavie and Fox, 2000; Lavie and de Fockert, 2003, 2005; MacDonald and Lavie, 2008; Torralbo and Beck, 2008; Benoni and Tsal, 2010; Caparos and Linnell, 2010; Elliott and Giesbrecht, 2010; Gaspelin et al., 2012; Roper et al., 2013). Brief exposure durations were originally adopted to preclude eye movements putatively as a means to disentangle overt from covert attentional processes (Lavie and Cox, 1997). The practice of brief exposure duration in load tasks deviates from the original flanker interference studies from which load theory is drawn as those pioneering studies employed display durations of 1000 ms (Eriksen and Hoffman, 1973; Eriksen and Eriksen, 1974). The use of limited display durations is not problematic *per se*, but failing to adequately recognize the role of exposure duration can be misleading when interpreting load theory.

On the assumption that brief exposure durations impose encoding demands, we manipulated the exposure duration of the stimulus array in a typical *p-*Load paradigm. We hypothesized that if the flanker effect solely depends upon display complexity (i.e., *p-*Load), then indefinitely extending the exposure duration, thereby reducing encoding demands, should have no bearing on the behavioral outcome (i.e., no flanker effect would be present under high *p-*Load). We orthogonally manipulated *p-*Load (canonical low *p-*Load vs. high *p-*Load) and exposure duration (100 ms vs. response-terminated). If temporal demands play no role in “spill-over,” then we expect to observe a robust load effect – flanker effect under low but not high *p-*Load. However, if temporal demands prevent robust flanker processing under high *p-*Load, then extending the exposure duration will increase the opportunity to sample the flanker thereby promoting a flanker effect.

To preview our results, we find a significant flanker effect under high *p-*Load when displays are response terminated. Experiment 1 suggests that brief exposure duration imposes encoding restrictions that manifest as the load effect. In Experiment 2, we orthogonally manipulated exposure duration and task-relevance by briefly presenting the target array (Experiment 2a) and the flanker (Experiment 2b). We found that, under high *p-*Load, interference effects are abolished when the target array is briefly displayed, but not when the temporal restriction falls on the flanker alone. As hypothesized by load theory, task-relevance must be considered to accurately describe how feature competition and temporal demands interact to engage selective attention.

## EXPERIMENT 1

### METHOD

#### Observers

Twenty-four University of Iowa undergraduates participated for course credit. All had normal or corrected-to-normal vision.

#### Apparatus

An Apple Mac Mini computer displayed stimuli on a 17-inch CRT monitor and recorded keyboard responses and latencies. The experiment was controlled using MATLAB and the Psychophysics toolbox (Brainard, 1997). Observers were seated 60 cm from the monitor.

#### Stimuli and procedure

The stimuli and procedure were modeled after Lavie’s (1995) load experiments. A target letter, equally likely to be X or Z, was randomly positioned in a six-item linear array (see **Figure 1**). The target was accompanied by five non-target letters which occupied the remaining positions along the horizontal. The letters K, V, S, J, and R comprised the high *p-*Load non-target set and the letter O, repeated five times, comprised the low *p-*Load, non-target set. The target and non-target letters were presented in uppercase, Helvetica font. They subtended a visual angle of 1.79° vertically and 1.55° horizontally and were separated by 0.60° (edge-to-edge). On every trial, a task-irrelevant flanker appeared 5.80° above or below the linear target array. The flanker was cortically magnified and subtended a visual angle of 2.84° vertically and 2.48° horizontally. Observers were encouraged to ignore the flanker. All stimuli were presented in black on a white background.

Observers were instructed to respond to the target via keyboard button press as quickly and accurately as possible. Observers pressed the “Z” key with the index finger one hand and the “?” key with the other index finger to indicate whether the target was Z or X. The flanker was equally likely to be response congruent (Z when the target was Z, likewise for the X), incongruent (X when the target was Z, or vice versa), or neutral (P which was not associated with any response).

**FIGURE 2:**
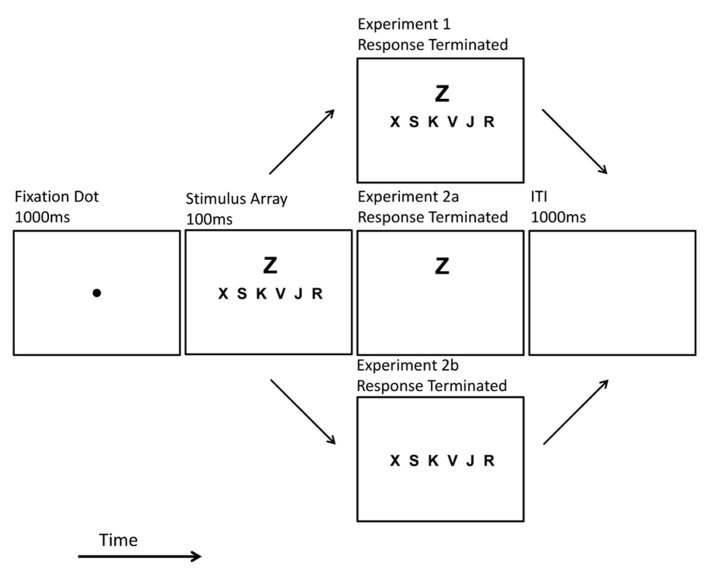
**Trial schematic.** Example of high *p-*Load, response terminated displays as presented in Experiments 1 and 2.

Each trial began with a central black fixation dot that appeared for 1000 ms. The target array and the flanker immediately replaced the fixation dot (see **Figure 2**). These stimuli were briefly displayed (100 ms) or remained until observer response. This exposure duration manipulation (brief or response-terminated) was blocked along with *p-*Load (high or low) thereby creating four unique block types. Block order was counterbalanced to produce 24 unique versions of the experiment (4! = 24). Each observer completed four blocks of 98 trials. Prior to the experiment, observers completed a 32-trial practice block. The results of the practice blocks were excluded from analyses.

### RESULTS AND DISCUSSION

**FIGURE 3:**
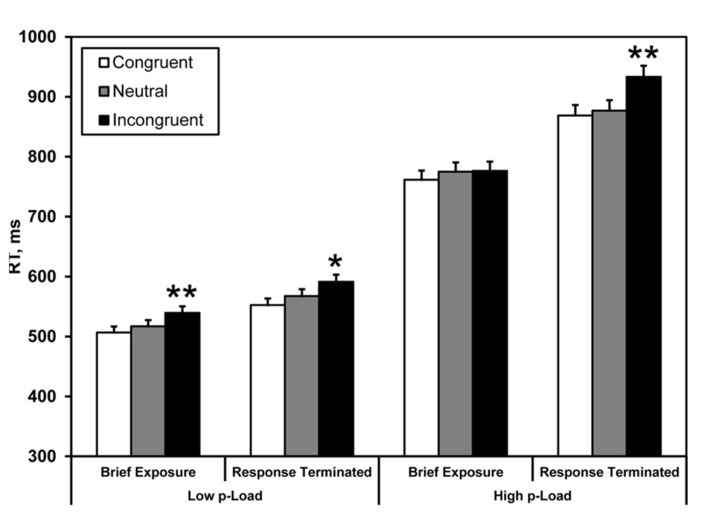
**Mean correct RT in Experiment 1.** Briefly exposed stimuli were presented for 100 ms – all other stimuli were response-terminated. Asterisks denote significance from neutral condition: *p < 0.05, **p < 0.01. Error bars represent 95% within-observers confidence intervals (Loftus and Masson, 1994; Morey, 2008).

Mean correct RTs were computed for each observer as a function of load, exposure duration, and the nature of the critical distractor (congruent, neutral, and incongruent). Response latencies ± 2.5 SD from the individual means were excluded from the analysis (see **Figure 3**); this trimming excluded 2.8% of the data.

A three-factor repeated measures ANOVA (2 × 2 × 2) was conducted on the RT data, with display *p-*Load (high vs. low), *m-*Load (100 ms vs. response-terminated), and flanker congruency (neutral vs. incongruent) as factors. We observed a main effect of *p-*Load, *F*(1, 23) = 119.44, *p* < 0.0005, $\eta_{p}^{2}$ = 0.84. RT was faster for low *p-*Load (*Mean* = *M* = 554 ms) than high *p-*Load (*M* = 840 ms). We also observed a main effect of *m-*Load, *F*(1, 23) = 11.76, *p* = 0.002, $\eta_{p}^{2}$ = 0.34. RT was faster when the display was briefly exposed (*M* = 652 ms) than when it was response-terminated (*M* = 742 ms). Additionally, we observed a significant main effect of flanker congruency, *F*(1, 23) = 34.4, *p* < 0.0005, $\eta_{p}^{2}$ = 0.60. RT was faster in the neutral condition (*M* = 684 ms) than in the incongruent condition (*M* = 710 ms). Most important, we observed a significant three-way interaction, *F*(1, 23) = 4.39, *p* = 0.047, $\eta_{p}^{2}$ = 0.16, and a significant two-way interaction between *m-*Load and congruency, *F*(1, 23) = 6.26, *p* = 0.020, $\eta_{p}^{2}$ = 0.21 which align our results with Lavie’s original finding (1995).

Accuracy performance paralleled RT effects (see **Table 1**). A three-factor repeated measures ANOVA (2 × 2 × 2) was conducted on accuracy data, with display *p-*Load (high vs. low), *m-*Load (100 ms vs. response-terminated), and flanker congruency (neutral vs. incongruent) as factors. We observed a main effect of *p-*Load, *F*(1, 23) = 29.39, *p* < 0.0005, $\eta_{p}^{2}$ = 0.56. Accuracy was better for low *p-*Load (*M* = 0.95, SE = 0.005) than high *p-*Load (*M* = 0.91, SE = 0.008). We also observed a main effect of *m-*Load, *F*(1, 23) = 68.77, *p* < 0.0005, $\eta_{p}^{2}$ = 0.75. Accuracy was better when the display was response terminated (*M* = 0.96, SE = 0.006) than when it was brief (*M* = 0.90, SE = 0.007). Additionally, we observed a significant main effect of flanker congruency, *F*(1, 23) = 33.83, *p* < 0.0005, $\eta_{p}^{2}$ = 0.60. Accuracy was better in the neutral condition (*M* = 0.94, SE = 0.005) than in the incongruent condition (*M* = 0.92, SE** = 0.006). We observed a significant two-way interaction, *F*(1, 23) = 29.66, *p* < 0.0005, $\eta_{p}^{2}$ = 0.56. No other comparisons reached significance.

**Table 1:** Experiment 1: accuracy performance.

		Flanker type									
		Incongruent		Neutral		Congruent	
		*M*	SD	*M*	SD	*M*	SD
Low load	Brief display	0.93	0.05	0.96	0.04	0.96	0.04
	Resp. term.	0.95	0.04	0.97	0.02	0.97	0.02
High load	Brief display	0.85	0.07	0.87	0.06	0.89	0.06
	Resp. term.	0.95	0.04	0.96	0.03	0.97	0.03

The results of the brief display condition replicated the load effect in that we observed a significant flanker effect for low but not high *p-*Load. The response-terminated displays produced a different pattern of results. Here we observed flanker effects irrespective of *p-*Load, indicating that display complexity interacts with temporal demands to produce the load effect.

This finding shares affinity with Miller’s (1991) assertion that even with fairly high *p-*Load, unattended flankers are processed semantically to some degree. To support his claim, Miller, 1991, Experiment 9) measured observers’ RT to identify a target letter in a heterogeneous display. Miller sought to delay the recognition of the flankers with respect to the target by presenting the target array slightly before the flankers. The flankers were presented in the periphery after a variable stimulus onset asynchrony (SOA) of 250, 350, and 450 ms. Significant flanker effects obtained regardless of SOA. Importantly, Miller used response terminated displays. The current findings reconcile the discrepancy between Miller’s load manipulation and those typically used to study *p*-Load on attention (e.g., Lavie, 1995).

Whereas Experiment 1 explored the demands marshaled by whole-display temporal constraints, Experiments 2a and 2b were designed to address whether *task-relevance* interacts with the temporal demands of the display to produce “spill-over.” Stimulus *relevance* has been critical to load theory’s interpretation since its inception (Lavie and Tsal, 1994). Only when the relevant task *p*-Load is low, will surplus attentional “resources” mandatorily “spill-over.” Our account makes the strong prediction that the task-relevant array’s exposure duration is most critical to witness load effects. When presented briefly, attention samples the task-relevant region at the expense of the flanker provided that the feature-based demands are sufficiently great. This leads to the natural prediction that briefly presenting the target array while leaving the flanker present until response will abolish flanker effects. This sort of prediction is at great odds with intuition. It seems irrational to expect the flanker to show no influence on behavior when it is present on-screen for the entire duration of the trial and is the only on-screen stimulus 100 ms into the trial. We designed Experiment 2a to test this hypothesis and find that when encoding demands are placed on the task-relevant array but not the flanker, “spill-over” is prevented despite the near-certain probability that the flanker was visibly isolated.

## EXPERIMENT 2

In Experiment 2a the target array offset after 100 ms, but the flanker persisted until response. We employed the converse relationship in Experiment 2b – the flanker offset after 100 ms, but the target array persisted until response.

### METHOD

The method of Experiment 2a was identical to Experiment 1 except that the offset of the target array and the flanker were asynchronous. Thus, in lieu of categorical response terminated displays such as those in Experiment 1, we introduce hybrid displays in Experiment 2 where 100 ms after stimulus onset, either the task-relevant or task-irrelevant portion of the display was removed – the lave being response terminated. Whereas Experiment 2a was characterized by the asynchronous removal of the target array, Experiment 2b was characterized by the asynchronous removal of the flanker. All stimuli were removed without backward masks. Observers were 48 (24 per experiment) University of Iowa undergraduates. All observers reported normal or corrected-to-normal vision. Experiments 2a and 2b were analyzed separately.

### RESULTS AND DISCUSSION

#### Experiment 2a

**FIGURE 4:**
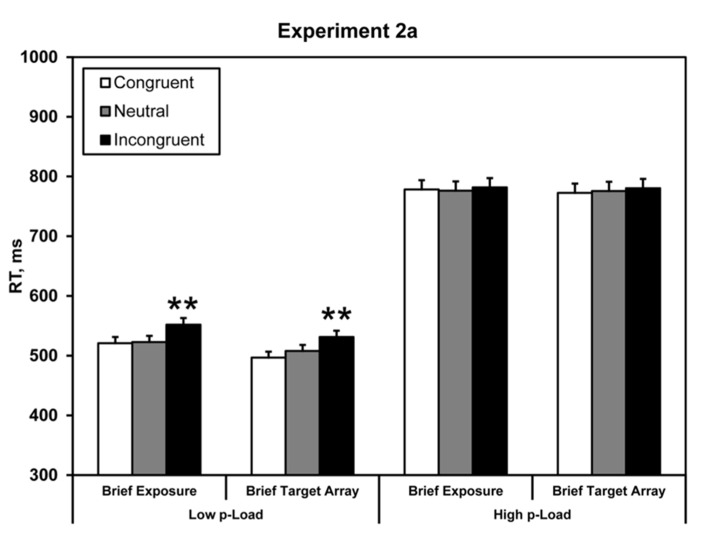
**Mean correct RT in Experiment 2a.** Briefly exposed stimuli were presented for 100 ms – all other stimuli were response-terminated. Asterisks denote significance from neutral condition: **p < 0.01. Error bars represent 95% within-observers confidence intervals (Loftus and Masson, 1994; Morey, 2008).

Mean correct RTs were computed for each observer as a function of *p-*Load, brief exposure (whole display or hybrid), and the nature of the critical distractor (congruent, neutral, and incongruent). Response latencies ± 2.5 SD from the individual means were excluded from the analysis (see **Figure 4**); this trimming eliminated 2.9% of the data.

Response time data were analyzed identically to Experiment 1 by carrying out a 2 × 2 × 2 repeated-measures ANOVA. We observed a main effect of load, *F*(1, 23) = 235.60, *p* < 0.0005, $\eta_{p}^{2}$ = 0.91. RT was faster under low *p-*Load (*M* = 528 ms) than high *p-*Load (*M* = 778 ms). Additionally, we observed a significant main effect of flanker congruency, *F*(1, 23) = 6.18, *p* = 0.021, $\eta_{p}^{2}$ = 0.21. Neutral RT (*M* = 646 ms) was faster than incongruent RT (*M* = 661 ms). The two-way interaction between *p-*Load and congruency was marginally significant, *F*(1, 23) = 4.20, *p* = 0.052, $\eta_{p}^{2}$ = 0.15.

Accuracy performance paralleled RT effects (see **Table 2**). A three-factor repeated measures ANOVA (2 × 2 × 2) was conducted on accuracy data, with display *p-*Load (high vs. low), *m-*Load (100 ms vs. response-terminated), and flanker congruency (neutral vs. incongruent) as factors. We observed a main effect of *p-*Load, *F*(1, 23) = 28.08, *p* < 0.0005, $\eta_{p}^{2}$ = 0.55. Accuracy was better for low *p-*Load (*M* = 0.95, SE = 0.007) than high *p-*Load (*M* = 0.90, SE = 0.010). We also observed a main effect of brief flanker, *F*(1, 23) = 28.88, *p* < 0.0005, $\eta_{p}^{2}$ = 0.56. Accuracy was better when just the flanker was brief (*M* = 0.95, SE = 0.006) than when the entire display was brief (*M* = 0.89, SE = 0.012). Additionally, we observed a significant main effect of flanker congruency, *F*(1, 23) = 15.00, *p* = 0.001, $\eta_{p}^{2}$ = 0.40. Accuracy was better in the neutral condition (*M* = 0.93, SE = 0.007) than in the incongruent condition (*M* = 0.91, SE = 0.008). We observed a significant two-way interaction, *F*(1, 23) = 27.73, *p* < 0.0005, $\eta_{p}^{2}$ = 0.5. No other comparisons reached significance.

**Table 2:** Experiment 2: accuracy performance.

			Flanker type									
			Incongruent		Neutral		Congruent	
			*M*	SD	*M*	SD	*M*	SD
Exp. 2a	Low load	Brief display	0.93	0.05	0.95	0.03	0.97	0.03
		Brief Flanker	0.94	0.06	0.96	0.03	0.96	0.02
	High load	Brief display	0.83	0.09	0.85	0.11	0.85	0.12
		Brief Flanker	0.95	0.04	0.96	0.03	0.95	0.05
Exp. 2b	Low load	Brief display	0.94	0.05	0.96	0.04	0.95	0.05
		Brief target	0.93	0.05	0.96	0.04	0.97	0.02
	High load	Brief display	0.84	0.08	0.88	0.06	0.87	0.06
		Brief target	0.93	0.09	0.95	0.09	0.94	0.08

Experiment 2a demonstrated that when the task-relevant array is temporally constrained, the flanker fails to exert a behavioral effect despite being the only on-screen stimulus for the majority of the trial. Thus, the load effect is closely linked with the exposure duration of the target array, not the flanker. Although the flanker lingered until response, it failed to exert an interference effect. We reason that briefly presenting the target imposes a mnemonic load (*m-*Load) on processing. This *m*-Load consumes mnemonic resources to endogenously represent the flanker thereby precluding robust flanker processing.

We take it as fact that the absence of a significant interference effect means that the flanker’s identity did not reach response selection. However, we cannot know for certain where the system ceased processing the flanker during its initial sweep. If we assume that the flanker needs to be consolidated into visual short-term memory (VSTM) before it can exert a downstream effect, then the absence of a downstream effect suggests two possibilities: (1) limited attentional “resources” initially deploy to preserve the representation of the fleeting task-relevant region at the cost of the task-irrelevant region, and (2) the flanker’s representation simply decays too fast. Experiment 2a falsifies the second possibility. If the flanker doesn’t reach response selection because it simply decays, then when we prevent decay by leaving it on-screen until response we ought to expect a flanker effect irrespective of any feature or encoding demand placed on the task-relevant array. However, we observed the contrary. Thus, in order for robust flanker processing all other encoding restrictions must be mitigated. Therefore, the flanker effect does not reside with the duration of the flanker. Furthermore, when we shorten its longevity but leave the task-relevant array until response, we should expect to obtain the canonical load effect. Experiment 2b was designed to test this hypothesis.

#### Experiment 2b

**FIGURE 5:**
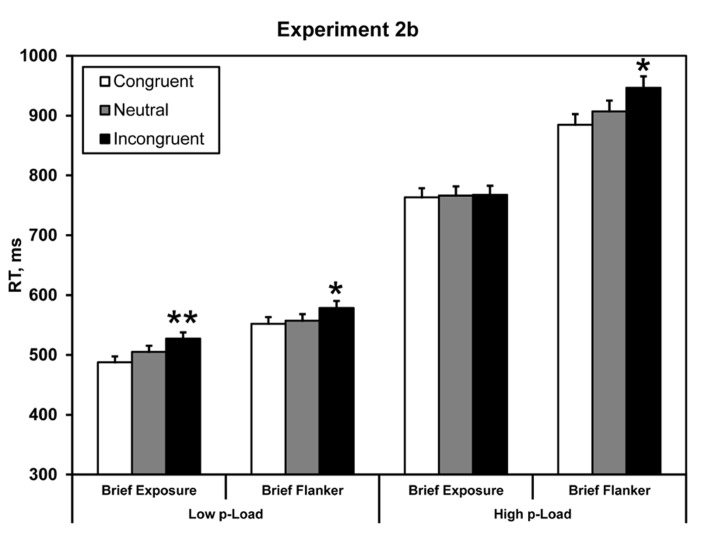
**Mean correct RT in Experiment 2b.** Briefly exposed stimuli were presented for 100 ms – all other stimuli were response-terminated. Asterisks denote significance from neutral condition: *p < 0.05, **p < 0.01. Error bars represent 95% within-observers confidence intervals (Loftus and Masson, 1994; Morey, 2008).

Mean correct RTs were computed for each observer as a function of *p-*Load, brief exposure (whole display or hybrid), and the nature of the critical distractor (congruent, neutral, and incongruent). Response latencies ± 2.5 SD from the individual means were excluded from the analysis (see **Figure 5**); this trimming eliminated 2.9% of the data.

Response time data were analyzed identically to the previous experiments by carrying out a 2 × 2 × 2 repeated-measures ANOVA. We observed a main effect of load, *F*(1, 23) = 164.98, *p* < 0.0005, $\eta_{p}^{2}$ = 0.88. RT was faster for low *p-*Load (*M* = 542 ms) than high *p-*Load (*M* = 847 ms). We also observed a main effect of target array duration, *F*(1, 23) = 11.58, *p* = 0.002, $\eta_{p}^{2}$ = 0.34. RT was faster when the entire display was briefly presented (*M* = 642 ms) as opposed to the flanker alone (*M* = 747 ms). Additionally, we observed a significant main effect of flanker congruency, *F*(1, 23) = 11.66, *p* = 0.002, $\eta_{p}^{2}$ = 0.34. Neutral RT (*M* = 684 ms) was faster than incongruent RT (*M* = 705 ms). Most important, we observed a significant two-way interaction between *p-*Load and flanker congruency, *F*(1, 23) = 6.02, *p* = 0.022, $\eta_{p}^{2}$ = 0.21. These results replicate our findings from Experiment 1 and Lavie’s original perceptual load demonstration.

Accuracy performance paralleled RT effects (see **Table 2**). A three-factor repeated measures ANOVA (2 × 2 × 2) was conducted on accuracy data, with display *p-*Load (high vs. low), *m-*Load (100 ms vs. response-terminated), and flanker congruency (neutral vs. incongruent) as factors. We observed a main effect of *p-*Load, *F*(1, 23) = 11.70, *p* = 0.002, $\eta_{p}^{2}$ = 0.34. Accuracy was better for low *p-*Load (*M* = 0.95, SE = 0.006) than high *p-*Load (*M* = 0.89, SE = 0.017). We also observed a main effect of brief flanker, *F*(1, 23) = 9.96, *p* = 0.004, $\eta_{p}^{2}$ = 0.30. Accuracy was better when just the flanker was brief (*M* = 0.94, SE = 0.012) than when the entire display was brief (*M* = 0.89, SE = 0.013). Additionally, we observed a significant main effect of flanker congruency, *F*(1, 23) = 56.53, *p* < 0.0005, $\eta_{p}^{2}$ = 0.71. Accuracy was better in the neutral condition (*M* = 0.93, SE = 0.010) than in the incongruent condition (*M* = 0.91, SE = 0.010). We observed a significant two-way interaction, *F*(1, 23) = 10.05, *p* = 0.004, $\eta_{p}^{2}$ = 0.30. No other comparisons reached significance.

In Experiment 2b we obtained a significant flanker effect under high *p-*Load when the flanker was briefly presented but the target array lingered until response. Although the flanker was briefly presented it nevertheless exerted a downstream effect. We propose that when briefly presented, the flanker’s icon can be sampled provided that the search stimuli remain visible. This proposition conforms to Experiment 1 and 2a in that both high stimulus competition and great encoding demands interact to produce the load effect. By briefly presenting the flanker in Experiment 2b, we incrementally loaded mnemonic processing^1^. Because the majority of stimuli remained onscreen, however, spare resources were available to process the fleeting flanker.

Experiments 1 and 2 indicate that the load effect vanishes when observers are not given strict encoding demands. These results point to the visual system’s highly adaptive nature; when items are in jeopardy of decaying away, the system can optimize attentional allocation toward task-relevant stimuli at the expense of task-irrelevant stimuli. Lavie demonstrated that high *p-*Load induces this response. We have demonstrated that great encoding demands also engage selection. Thus, mnemonic “resource” limitations can serve as an additional processing bottleneck that activates selection and drives the load effect.

## GENERAL DISCUSSION

Historically, the load effect has been thought to be driven entirely by perceptual-level resource demands, not data limitations (Lavie and de Fockert, 2003); however, our experiments have shown that in fact both *p-*Load and *m-*Load play similar, but distinct roles in distractor processing. In Experiment 1, we extended the exposure duration of a canonical load task and demonstrated that when the encoding restrictions imposed by brief displays are lifted, flanker effects obtain despite high *p-*Load. In Experiment 2b, we demonstrated the importance of task-relevance when considering the influence that *m-*Load has on selective attention. We observed a significant interference effect when the flanker was briefly exposed but the task-relevant array remained until response. This finding starkly contrasts with Experiment 2a, the inverse situation, where the flanker remained until response but the task-relevant array was briefly exposed. In Experiment 2a, although the flanker remained on-screen, it failed to exert a behavioral effect under high *p-*Load. The results provide a possible compromise for load theory that incorporates *m-*Load and *p-*Load as potential restrictions that interact to set the locus of selective attention. We cannot observe *p*-Load effects without implementing an *m*-Load; conversely, we cannot observe *m*-Load effects without implementing a *p*-Load.

**FIGURE 6:**
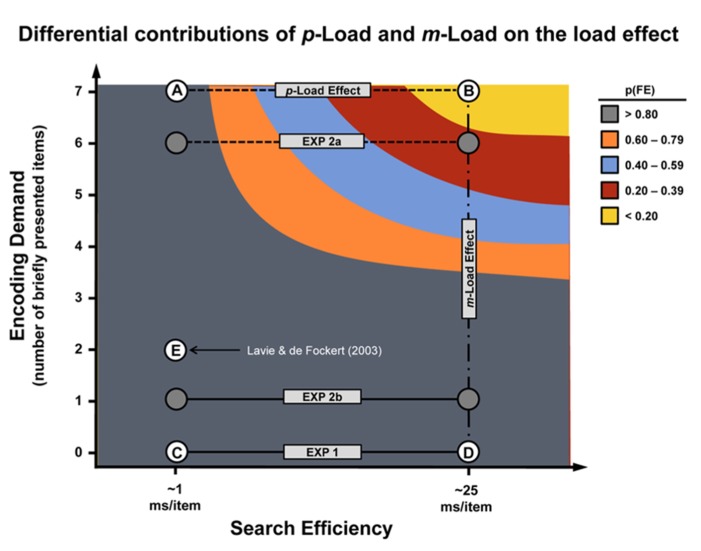
**Load effect “resources.”** This empirically derived model illustrates two forms of “resource” demand – feature- and encoding-based – that impinge upon selective attention to produce the load effect (denoted by dashed lines). The vertical axis denotes the *m*-Load and is quantified as the number of briefly exposed display stimuli. The horizontal axis denotes the *p-*Load and is quantified in terms of the search efficiency of the displays. These values are estimated based on our previous work exploring the role of visual search and load (Duncan and Humphreys, 1989; Roper et al., 2013). Points A and B represent the canonical low *p-*Load and high *p-*Load tasks, respectively. Tasks represented by point A and B served as control conditions for Experiments 1 and 2. Low *p-*Load was characterized by a conspicuous target (search efficiency ~1 ms/item), whereas high *p-*Load was characterized by an inconspicuous target (search efficiency ~25 ms/item) – both conditions are characterized by high *m-*Load (7 stimuli at 100 ms exposure). Experiments 2a and 2b, denoted by filled circles, were hybridized and fall between line segments AB and CD in terms of *m-*Load (see Experiment 2 methods). Point C represents a low *m-*Load, low *p-*Load task and point D represents a low *m-*Load, high *p-*Load task (both unique to Experiment 1). Point E reflects a high *m-*Load, low *p-*Load task (Experiment 2, Lavie and de Fockert, 2003). N.B., although feature competition and encoding demands can be manipulated orthogonally, it is not entirely clear whether these factors are psychologically independent. The shape of the curve is intended for demonstration only.

**Figure 6** highlights the individual contributions of *m-*Load and *p-*Load on distractor processing. Tasks with high *m-*Load (points A and B in **Figure 6**) but with low *p-*Load (point A) lead to significant flanker effects as has been shown previously (Lavie and Tsal, 1994). Experiment 1 demonstrated that tasks with high *p-*Load (points D and B in **Figure 6**) but without accompanying high *m-*Load (point D) also gives rise to significant flanker effects. Therefore adequate consideration must be given to *p-*Load and *m-*Load to fully capture the load effect. In Experiments 2a and 2b, we demonstrated that the *m-*Load needs to be placed on the task-relevant array, not the flanker, to obtain the load effect. We speculate that this is because there is more visual information that needs to be encoded when the six-item target array is briefly presented. These encoding demands are compounded when *p*-Load is great. As demonstrated in Experiment 2a, when mnemonic resources are preoccupied by the necessity to quickly encode the target array, the flanker fails to engender an interference effect even when it remains on-screen until response.

### SENSORY AND ENCODING DEMANDS

Lavie and de Fockert (2003) increased task difficulty by degrading the target stimulus and decreasing exposure duration (50 ms) in a low *p-*Load task. They argued that the flanker effect would be abolished only if the imposed data limitations play a role in selective attention. The data revealed a significant flanker effect, suggesting that data limitations do not drive *p-*Load. Such a conclusion runs counter to our current findings, but can be explained by carefully inspecting **Figure 6**. First, we assert that due to relatively low *p-*Load and 100 ms exposure duration, conventional low *p*-Load tasks fall near point A in **Figure 6**. From Norman and Bobrow (1975) we know that masked, degraded, and brief displays constitute data limitations. If these arguments hold, then it follows that the flanker task with a degraded target and very limited exposure duration as described above and tested by Lavie and de Fockert (2003) would deviate from point A in the vertical direction only. Our account predicts a significant flanker effect under these conditions. Therefore, we propose that the load effect arises from attentional resource demands imposed by the display’s perceptual characteristics coupled with the encoding demands introduced by brief stimulus presentation. Limiting the exposure duration prevents complete processing of all available stimuli due the severe capacity limitations inherent to memory consolidation and representation. When this happens, the least relevant stimuli are excluded from robust processing.

### SELECTIVE ATTENTION AND VISUAL SHORT-TERM MEMORY

In three experiments we have demonstrated that the load effect dissipates when the temporal demands to quickly encode the task-relevant stimuli are removed. Counter-intuitively, the likelihood of processing the task-irrelevant flanker does not reside in the longevity of the flanker itself but rather with the availability of attentional “resources.” These findings implicate VSTM capacity limitations as a substantial bottleneck for attentional processing.

Briefly presented stimuli place temporal demands on visual processing (Jolicœsur and Dell’ Acqua, 1999). Thus, fleeting environmental information must be internally represented before it reaches downstream processing. We propose that limited display durations increase temporal encoding demands. Exactly where along the stream of processing these demands exert the greatest effect is not precisely known; however, Roper and Vecera (2013) demonstrated that increments in VSTM load produced greater selective attention in a concurrently performed *p-*Load task. Konstantinou and Lavie (2013) replicated this effect in a standard selection task. Thus the reliance on VSTM to identify the target and program the correct response has been empirically founded. Furthermore, these two recent studies demonstrate that domain-specific *m-*Loads impede distractor processing but domain-general *m-*Loads exacerbate the distraction effect. Because VSTM capacity is spatially and temporally restricted (Zhang and Luck, 2008), when stimuli are too numerous or too complex, selective attention can prioritize entrance into VSTM based on task relevance. In the typical load task, the task-relevant search array appears around fixation, and the flanker appears at a location never occupied by a target. Observers can optimally search for the target by segregating the display based on task-relevance, allowing attention to prioritize likely target locations over unlikely target locations. We hypothesize that under high *p*-Load, observers prioritize potential target locations for entry into VSTM at the expense of the task-irrelevant flanker. Under low *p*-Load, ample VSTM capacity exists, and attention need not precisely prioritize entry into VSTM – in fact, based on the evidence, under low *p*-Load, all stimuli are mandatorily processed (Lavie, 1995). This account need only assume that the flanker be encoded in VSTM prior to exerting behavioral effects – a safe assumption given that access to VSTM satisfies a necessary, but not sufficient, condition to witness competition at the output stage.

This work converges with recent work by Konstantinou et al. (2012), who found that a concurrent VSTM load attenuated contrast sensitivity. Decreased brain metabolism in primary and tertiary visual areas (V1-V3) accompanied the contrast sensitivity decrement. Extending from Bundesen (1990) and Bundesen et al.’s (2005) Theory of Visual Attention (TVA), Kyllingsbæk et al. (2011) demonstrated that load effects are best explained by appealing to a model that incorporates processing capacity and VSTM capacity limits. Participants reported the identity of several targets in briefly presented displays while ignoring flanking letters. Target identification declined as the number of flankers increased, a result not readily predicted by load theory. Kyllingsbæk et al. (2011) argued TVA could readily explain their results – as the number of flankers increases, flankers are more likely to enter VSTM, which reduces the likelihood that a target will enter VSTM.

The previously described extant studies bolster the current work and provide direct tests to support our conclusion that the load effect relies upon the availability of VSTM resources. Thus it is reasonable to conclude that load, as it has been previously tested, is not exclusively perceptual but rather partly determined by mnemonic processing limitations. This assertion is based upon work that demonstrates that the bandwidth of mnemonic processing is limited, but the bandwidth of perceptual processing is virtually limitless (Sperling, 1960; Averbach and Coriell, 1961; Potter, 1976; Coltheart, 1980; Di Lollo, 1980; Jolicœsur and Dell’ Acqua, 1998; Vogel et al., 1998). These findings indicate that selective attention may play as vital a role in VSTM as perception (Schmidt et al., 2002).

### LOAD THEORY AND DILUTION

Tsal and Benoni (2010) have recently proposed that the load effect arises from diluting the display with additional neutral stimuli and not *p*-Load itself. To support their proposition, they carefully crafted a high-dilution display that was nevertheless low in *p*-Load. This was accomplished by placing neutral stimuli – that putatively dilute the flanker – at otherwise task-irrelevant locations. They hypothesized that if dilution, not *p*-Load, determines the locus of selection, then these high-dilution, low *p-*Load displays will fail to produce a flanker effect. Their predictions were confirmed; neutral but otherwise task-irrelevant distractors abolished the flanker effect. Like the load effect, the dilution effect is robust and has been replicated several times (Wilson et al., 2011; Benoni and Tsal, 2010, 2012).

The dilution alternative is incompatible with load theory; however, the current experiments are in line with both accounts. Although the current experiments were not designed to examine dilution some loose parallels can be drawn. It is entirely possible that dilution effects are experienced as *m*-Load. For instance, increments in display size may place demands on capacity restricted cognitive mechanisms like VSTM. When VSTM is loaded, the identity of the flanker becomes diluted in memory and thus fails to reach the response selection stage. How exactly dilution fits into this framework remains an open question, but acknowledging *m*-Load may provide an avenue to reconcile long-standing facets of load theory and the dilution account.

## CONCLUSION

To explain attentional “spill-over” phenomena, load theory invokes unclear resources. We classified these resources into two categories: processing demands stemming from (1) the need to resolve feature-based competition, and (2) the need to readily encode fleeting visual representations. We proposed that whereas attentional resources satisfy the former condition, free mnemonic resources satisfy the latter. We introduced the term *m*-Load to describe task-imposed encoding restrictions and we presented a new account that reflects how *m*-Load interacts with conventional feature-based *p*-Load to produce the load effect. Lastly, we suggested that *m*-Load may prevent the flanker effect by denying the flanker’s entry into VSTM. This contributes to the growing body of work on load theory and extends it to include two distinct classes of processing challenges set by the task environment.

## Conflict of Interest Statement

The authors declare that the research was conducted in the absence of any commercial or financial relationships that could be construed as a potential conflict of interest.

## Footnotes

1 We would like to stress that the flanker was not backward-masked in Experiment 2. Therefore, based on the presence of the flanker effect in high *p*-Load, it is likely that the identity of the flanker was obtained from the visual icon (Averbach and Coriell, 1961; Phillips, 1974). We reason that the visual system consolidated the flanker into VSTM to prevent complete decay. Indeed visual transients, such as abrupt-offsets, have been shown to capture attention (Pratt and McAuliffe, 2001), and attention-capturing stimuli are automatically consolidated in VSTM (Schmidt et al., 2002; Belopolsky et al., 2008). However, we acknowledge the possibility that the flanker circumvented VSTM, but this scenario is less likely. Our own investigations have revealed that when the flanker is backward-masked, canonical load effects obtain (Roper et al., 2011).
